# Impact of Preoperative Sarcopenia on Survival and Postoperative Outcomes in Esophageal Cancer Patients Undergoing Esophagectomy: A Single-Center Retrospective Study

**DOI:** 10.7759/cureus.77521

**Published:** 2025-01-16

**Authors:** Zachary K Woodward, Goutham Sivasuthan, Ratna Aseervatham, Priscilla Martin

**Affiliations:** 1 General Surgery, Sunshine Coast University Hospital, Birtinya, AUS

**Keywords:** ct sarcopenia, esophageal cancer, esophagectomy, sarcopenia, sarcopenic, skeletal muscle index

## Abstract

Background

Sarcopenia is the progressive and generalized loss of skeletal muscle and its associated function. Whilst it is typically associated with advanced age, it is also prevalent in patients with chronic diseases including cancer. Patients with esophageal cancer are at high risk of developing malnutrition and sarcopenia due to impaired oral intake, the effects of neoadjuvant treatment, and cancer-related cachexia. Sarcopenia has been associated with worse postoperative outcomes. The aim of this study was to investigate the impacts sarcopenia had on the short- and long-term outcomes of patients undergoing esophagectomy in a regional Australian context.

Methods

A single-center retrospective analysis was performed for 48 patients who underwent esophagectomy, most of which were for esophageal cancer. All eligible patients received neoadjuvant treatment prior to surgery. Patients were classified as sarcopenic based on their calculated skeletal muscle index (SMI) on a preoperative computed tomography scan. SMI criteria for sarcopenia were <52.4 cm^2^/m^2^ for males and <38.5 cm^2^/m^2^ for females. Outcomes measured included overall and disease-free survival, postoperative complications, and length of hospital stay.

Results

Of the 44 patients who met inclusion criteria and underwent esophagectomy, 27 were sarcopenic based on preoperative computed tomography skeletal muscle measurements at L3. The average overall survival for the sarcopenic group was 20.1 months (95% CI 13.3-26.9) with a one-, two-, and three-year overall survival rate of 59.3%, 29.6%, and 22.2%, respectively. The non-sarcopenic group had an average overall survival rate of 28.8 months (95% CI 19.6-38.1) with a one-, two-, and three-year overall survival rate of 82.4%, 41.2%, and 29.4%, respectively. The average disease-free survival for the sarcopenic group was 14.1 months (95% CI 8.4-19.8) with a one-, two-, and three-year disease-free survival rate of 37.0%, 18.5%, and 11.1%, respectively. The average disease-free survival rate for the non-sarcopenic group was 27.2 months (95% CI 19.7-34.7) with a one-, two-, and three-year disease-free survival rate of 76.5%, 41.2%, and 29.4%, respectively. The sarcopenic group had an increased average length of hospital stay (23.9 days (CI 95% 16.5-31.3) vs. 14.6 days (95% CI 12.2-17.0)). A higher proportion of the sarcopenic patients had restricted dietary intake and required either pureed or enteral feeding (36% vs. 9%). No difference in postoperative complications was detected between the groups.

Conclusions

Patients with preoperative sarcopenia had a lower overall and disease-free survival and an increased length of hospital stay when compared with non-sarcopenic patients. Additionally, sarcopenic patients had a higher likelihood of requiring pureed or enteral feeds preoperatively.

## Introduction

Esophageal cancer carries a significant burden of disease; globally, it is the 11th most common cancer by incidence and ranks 7th in terms of mortality [1]. For tumors located within the esophagus or at the gastroesophageal junction, the gold standard treatment is esophagectomy.

Esophagectomy is a major surgical operation with a relatively high risk of morbidity and mortality. Perioperative complications include anastomotic leak, chyle leak, recurrent laryngeal nerve injury, and pneumonia [2]. The postoperative morbidity has been estimated to be between 25% and 47%, whilst the perioperative mortality rate is between 1.7% and 4% [3,4].

Histologically, esophageal cancer is typically categorized as adenocarcinoma or squamous cell carcinoma. Adenocarcinoma is the most predominant type in North America, Europe, and Oceania, while squamous cell carcinoma predominates in Asia [5].

Patients with esophageal cancer typically present malnourished due to inadequate nutrition due to swallowing difficulty as the tumor may partially or completely obstruct the esophagus [6]. Additionally, the cancer can induce chronic systemic inflammation and a hypermetabolic state, which further contributes to weight loss and cachexia [7]. These effects are compounded in patients who receive neoadjuvant therapy, which has been shown independently to reduce skeletal muscle mass [8,9].

Sarcopenia is defined as a reduction in skeletal muscle mass accompanied by a loss of function [10]. Importantly, sarcopenia is not limited to thin patients, as overweight or obese persons may also have reduced muscle mass. For esophageal cancer, between 26% and 75% of patients will be sarcopenic at the time of initial presentation, depending upon the stage of the disease [11]. Previous studies have shown that preoperative sarcopenia is associated with a higher risk of perioperative complications in patients undergoing esophagectomy and reduced long-term survival [12,13].

As relatively few studies have been conducted that have investigated the role sarcopenia has on esophagectomy patients in a regional Australian context, this study aimed to examine the short- and long-term outcomes of sarcopenic patients undergoing esophagectomy in a regional Australian hospital.

## Materials and methods

Patient selection

A single-center retrospective analysis was undertaken of patients who underwent an esophagectomy between January 2019 and December 2024 at the Sunshine Coast University Hospital, Australia. Patients who had an esophageal or gastroesophageal junction cancer and underwent an esophagectomy were included. Patients who underwent esophagectomy for non-malignant pathology were excluded. Medical records were reviewed for patient demographics, comorbidities, pathological staging, survival outcomes, and disease recurrence. Nutritional information including diet, albumin, and preoperative hemoglobin was recorded. Patients were considered to be on a restricted diet if they required pureed or enteral feeds.

All eligible patients received neoadjuvant therapy prior to surgery following the ChemoRadiotherapy for Oesophageal cancer followed by Surgery Study (CROSS) [14] or fluorouracil, leucovorin, oxaliplatin, and docetaxel (FLOT) protocol [15]. Patients typically proceeded to surgery within six weeks after completing neoadjuvant therapy. Depending on tumor location, patients underwent either an Ivor Lewis or McKeown esophagectomy. Ivor Lewis esophagectomy was performed via laparotomy and thoracotomy. McKeown esophagectomy was performed via laparotomy, thoracotomy, and neck incision. The neo-esophagus was typically formed from the stomach and anastomosed to the proximal esophagus via the posterior mediastinum. A D1 lymphadenectomy was performed in both operations for nodal assessment. Pyloroplasty, placement of a feeding jejunostomy tube, and the placement of abdominal and chest drains were also routine in both types of operation. All patients were initially managed in the intensive care unit postoperatively.

Postoperative complications were defined as described by the International Consensus on Standardization of Data Collection for Complications Associated With Esophagectomy [2] and were classified according to the Clavien-Dindo classification of surgical complications [16]. Postoperative complications were divided into respiratory, cardiac, urological, thromboembolic, wound infection, delirium, bleeding, chyle leak, and anastomotic leak. Any complications that required readmission to the hospital within 30 days of discharge were also recorded. Disease-free survival was measured from the date of operation until disease recurrence was confirmed on repeat imaging.

Measurement of sarcopenia

**Figure 1:**
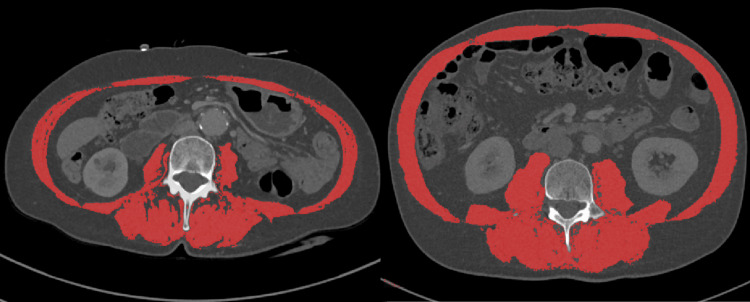
Example of a CT from a sarcopenic patient (left) and a non-sarcopenic patient (right). Highlighted area is the measured skeletal cross sectional area used to calculate the skeletal muscle index

Sarcopenia was measured on a preoperative computed tomography (CT) scan using the methodology outlined by Gomez-Perez et al. [17]. All CT scans analyzed were performed prior to the commencement of neoadjuvant therapy or immediately prior to surgery for patients who did not undergo neoadjuvant treatment. The cross-sectional area of the skeletal muscle was measured on a single axial CT image slice at the level of the mid-L3 vertebra using ImageJ (National Institutes of Health, Bethesda, USA), a public-domain imaging processing software. After attenuation thresholds were set between −29 and +150 Hounsfield units, the inner and outer perimeter of the paraspinal and abdominal wall musculature was traced to give the skeletal muscle cross-sectional area, displayed as the highlighted area in Figure 1. The skeletal muscle area was subsequently divided by the patient’s height squared to give the skeletal muscle index (SMI).

$\text{Skeletal Muscle Index }(cm^{2}/m^{2}) = \frac{\text{Muscular Cross Sectional Area }(cm^2)}{\text{Height }(m^2)}$

Patients with an SMI below 52.4 cm^2^/m^2^ for males and 38.5 cm^2^/m^2^ for females were considered sarcopenic.

Statistical analysis

Statistical analysis was performed with IBM SPSS Statistics for Windows, Version 26 (Released 2019; IBM Corp., Armonk, New York, United States). Patients were categorized as sarcopenic or non-sarcopenic based on preoperative CT scans; these groups were subsequently compared. Continuous variables were analyzed with an unpaired two-tailed t-test, whilst categorical data was compared with a chi-squared test. Survival curves were plotted using the Kaplan-Meier method, and differences were analyzed with a log-rank test. For the disease-free survival curve, patients who died were censored. A p-value of <0.05 was considered significant.

Ethics approval

Ethics approval for the study was granted by the Metro South Health Human Research Ethics Committee (HREC/2024/QMS/109200). Site-specific approval was granted by the Sunshine Coast University Hospital Office for Research Governance and Development (QSC/109200).

## Results

Patient information

Between January 2019 and December 2024, 48 esophagectomies were performed at the Sunshine Coast Hospital, four of which were not included in this study due to non-malignant pathology. The remaining study population was 89% male, and the average age was 67 years (range: 28-82 years); there was no significant age difference between the sarcopenic and non-sarcopenic groups. Sarcopenia was present in 61.4% of participants based on CT analysis. The average follow-up time for all participants was 25 months (95% CI, 19.0-30.1, range: 2-66 months). Patient demographics, comorbidities, and nutritional information are presented in Table 1. Sarcopenic patients had a significantly lower average weight (70.2 kg vs. 82.6 kg, p = 0.006) and BMI (23.3 kg/m^2^ vs. 27.8 kg/m^2^, p < 0.001) than the non-sarcopenic group. The sarcopenic group was also more likely to have restricted dietary intake (36% vs. 9%, p = 0.020), which required either pureed or enteral feeding for nutrition. All patients received neoadjuvant therapy except three within the sarcopenic group. One patient was deemed ineligible for neoadjuvant treatment due to a pancreatic collection, another was excluded as they had previously undergone neoadjuvant treatment for a different cancer, and the last had an emergent presentation with an esophageal perforation. There were no significant differences between the groups in terms of comorbidities, type of surgery, or neoadjuvant treatment. Preoperative hemoglobin and albumin were similar between the groups.

**Table 1:** Comparison of demographic, comorbidity, and treatment data between sarcopenic and non-sarcopenic groups a: unpaired two-tailed t-test; b: chi-squared test CABG: coronary artery bypass graft; CVD: cerebrovascular disease; TIA: transient ischemic attack; COPD: chronic obstructive pulmonary disease; BMI: body mass index

Factor	Sarcopenic (n = 27)	Non-sarcopenic (n = 17)	p-value	Test
Age (years)	68.5 ± 10.6	64.1 ± 9.7	0.169	a
Gender				
Male	24	15	0.947	b
Female	3	2		
Comorbidities				
Diabetes	3	1	0.557	b
Hypertension	16	6	0.122	b
Dyslipidemia	6	3	0.714	b
Ischemic heart disease	1	2	0.302	b
CABG	1	0	0.422	b
Pacemaker	1	1	0.736	b
Atrial fibrillation	2	2	0.624	b
CVD/TIA	2	1	0.845	b
Renal impairment	2	0	0.251	b
Vascular disease	1	2	0.302	b
COPD	2	2	0.625	b
Smoker or ex-smoker	18	14	0.255	b
Pack years	27.6 ± 16.3	27.0 ± 13.2	0.911	a
Alcohol (standard drinks/day)	1.7 ± 2.6	1.2 ± 2.5	0.484	a
Nutrition				
Height (cm)	173.4 ± 7.2	171.8 ± 7.5	0.482	a
Weight (kg)	70.2 ± 11.0	82.6 ± 14.9	0.006	a
BMI (kg/m^2^)	23.3 ± 3.4	27.8 ± 3.9	<0.001	a
Restricted diet	16	4	0.020	b
Hemoglobin (g/L)	122.3 ± 14.0	126.4 ± 13.7	0.339	a
Albumin (g/L)	33.9 ± 5.4	33.6 ± 4.9	0.833	a
CT measurements				
Skeletal muscle area (cm^2^)	137.2 ± 22.4	167.0 ± 22.2	<0.001	a
Skeletal muscle index (cm^2^/m^2^)	45.4 ± 5.5	56.3 ± 4.2	<0.001	a
Surgery				
Ivor-Lewis	22	15	0.550	b
McKeown	5	2		
Neoadjuvant therapy	24	17	0.155	b
CROSS	23	15	0.774	b
FLOT	1	2	0.301	b

Histology

There were no significant differences between the sarcopenic and non-sarcopenic groups with regard to tumor location, tumor type, histologic grade, TNM staging, or resection margins. Adenocarcinoma was the most common tumor type, accounting for 84.1% of the cohort overall. For the sarcopenic and non-sarcopenic groups, adenocarcinoma accounted for 47.7% and 36.4%, respectively. Of the six squamous cell carcinomas, five were found in the sarcopenic group (11.4%), whilst one was in the non-sarcopenic group (2.3%). One patient within the sarcopenic group had a poorly differentiated carcinoma that could not be confidently categorized as either type. Although it did not reach statistical significance, a comparatively higher number of sarcopenic patients were found to have stage N1 disease. Histology data is reported in Table 2.

**Table 2:** Histologic data for sarcopenic and non-sarcopenic patients a: unpaired two-tailed t-test; b: chi-squared test

Specimen pathology	Sarcopenic (n = 27)	Non-sarcopenic (n = 17)	p-value	Test
Tumor location				
Mid esophagus	4	1	0.363	b
Distal esophagus	6	1	0.149	b
Gastro-esophageal junction	17	15	0.067	b
Histology				
Adenocarcinoma	21	16	0.217	b
Squamous cell carcinoma	5	1		
Tumor Grade			0.286	b
Well-differentiated	1	3	0.117	b
Moderately differentiated	14	7	0.490	b
Poorly differentiated	12	7	0.831	b
T staging				
T1	4	2	0.774	b
T2	5	3	0.942	b
T3	12	6	0.548	b
T4	1	2	0.302	b
Complete pathological response	5	3	0.942	b
N staging				
N0	14	11	0.703	b
N1	10	2	0.067	b
N2	2	3	0.298	b
N3	1	1	0.736	b
Mean nodes harvested	16 (range 6-33)	20 (range 6-38)	0.163	a
Mean nodes positive	1 (range 0-9)	1 (range 0-8)	0.742	a
Resection margin				
R0	23	16	0.363	b
R1	4	1		

Patient outcomes

Patient outcome information can be found in Table 3. The sarcopenic group had significantly longer hospital admissions, requiring on average 9.3 days longer in the hospital when compared with the non-sarcopenic group. There was no significant difference between the rates of other complications between the sarcopenic and non-sarcopenic groups, including for the rates of anastomotic leak and respiratory complications. While not statistically significant, the two patients who died within 30 days of their operation were in the sarcopenic group. Both had stormy postoperative courses, with one dying from complications of an anastomotic leak, whilst the other suffered unsurvivable bowel ischemia from a suspected arterial thrombus.

**Table 3:** Outcomes for sarcopenic and non-sarcopenic patients a: unpaired two-tailed t-test; b: chi-squared test AKI: acute kidney injury; UTI: urinary tract infection; PE: pulmonary embolism; DVT: deep vein thrombosis

Outcomes	Sarcopenic (n = 27)	Non-sarcopenic (n = 17)	p-value	Test
Length of admission (days)	23.9 ± 19.9	14.6 ± 5.3	0.028	a
Readmission (within 30 days)	5	3	0.942	b
Death (within 30 days)	2	0	0.251	b
Complications				
Respiratory	12	7	0.831	b
Cardiac	7	3	0.523	b
Urological (AKI, UTI)	2	3	0.297	b
Thromboembolic (PE/DVT)	4	0	0.096	b
Wound infection	2	0	0.251	b
Anastomotic leak	6	2	0.381	b
Bleeding	1	0	0.422	b
Chyle leak	3	1	0.557	b
Delirium	5	1	0.234	b
Clavien-Dindo classification				
≤2	22	13	0.689	b
≥3	12	7	0.624	b

Overall survival

**Figure 2:**
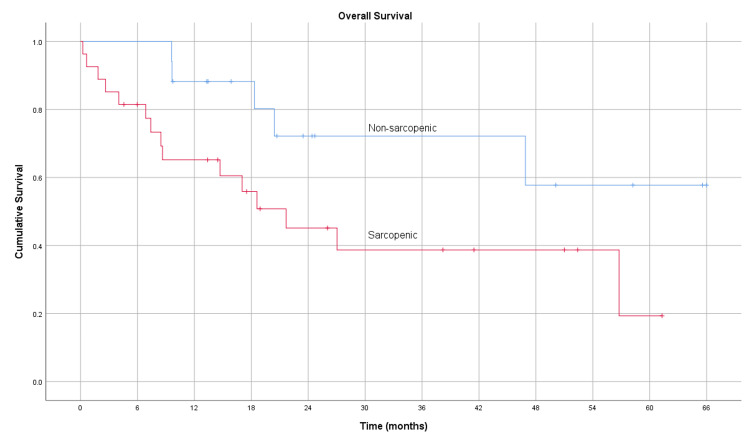
Kaplan-Meier curve for overall survival of 27 sarcopenic and 17 non-sarcopenic patients (p = 0.046)

The average overall survival for the sarcopenic group and non-sarcopenic groups was 20.1 months (95% CI 13.3-26.9) and 28.8 months (95% CI 19.6-38.1), respectively. The whole population's overall one-, two-, and three-year survival rates were 68.2%, 34.1%, and 25.0%, respectively. Overall survival was lower in the sarcopenic group for one-, two-, and three-year survival with 59.3%, 29.6%, and 22.2%, respectively. In the non-sarcopenic group, the one-, two-, and three-year survival rates were 82.4%, 41.2%, and 29.4%, respectively. The Kaplan-Meier curve for overall survival is depicted in Figure 2; the log-rank test showed a significant difference between the sarcopenic and non-sarcopenic (p = 0.046).

Disease-free survival

**Figure 3:**
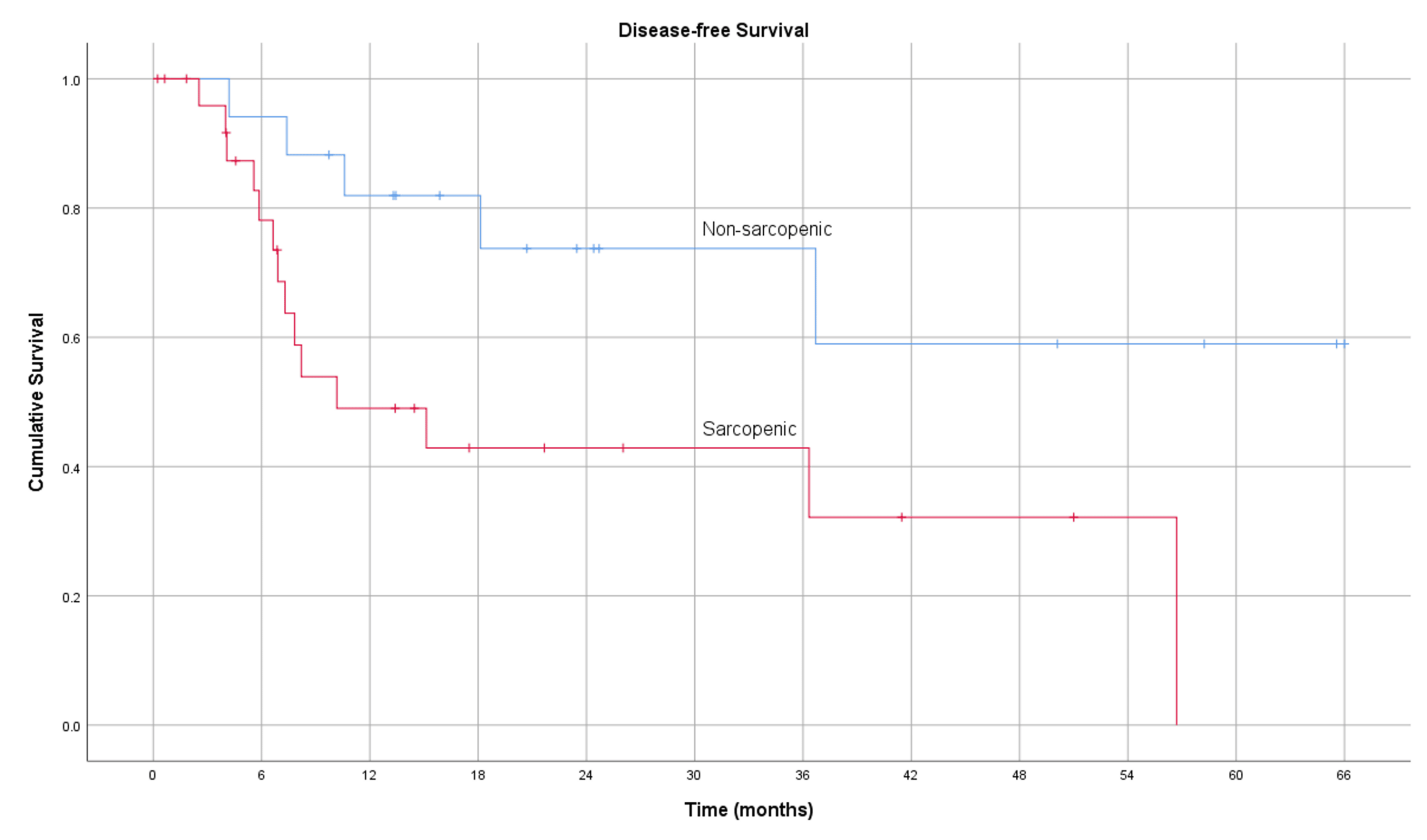
Kaplan-Meier curve for disease-free survival of 27 sarcopenic and 17 non-sarcopenic patients (p = 0.017)

The average disease-free survival for the sarcopenic and non-sarcopenic groups was 14.1 months (95% CI 8.4-19.8) and 27.2 months (95% CI 19.7-34.7), respectively. Whole cohort disease-free survival rates for one, two, and three years were 52.3%, 27.3%, and 18.2%, respectively. The disease-free survival rates in the non-sarcopenic group for one, two, and three years were 76.5%, 41.2%, and 29.4%, respectively. The disease-free survival for the sarcopenic group was lower, with one-, two-, and three-year rates of 37.0%, 18.5%, and 11.1%, respectively. The Kaplan-Meier curve for disease-free survival is depicted in Figure 3; the log-rank test showed a significant difference between the sarcopenic and non-sarcopenic groups (p = 0.017).

## Discussion

The rate of sarcopenia in previous studies varies considerably, with a range between 14.4% and 81.0% [9]; within this study, the rate of sarcopenia was 61.4%. The rate of sarcopenia differs substantially depending on the study population, age, neoadjuvant treatment, the method of diagnosing, and criteria for sarcopenia. There is currently no agreed-upon definition for CT-diagnosed sarcopenia; however, the criteria used in this study, as initially proposed by Prado et al. [18], have been utilized across multiple similar studies [11,19].

The sarcopenic group had both a lower overall survival rate and a lower disease-free survival rate when compared with the non-sarcopenic group, which is consistent with recent meta-analyses on the topic [12,20]. The association of sarcopenia with lower overall survival has been seen across many different cancer types, including colorectal cancer [21] and gastric cancer [22]. Although it did not reach statistical significance, the sarcopenic group showed a higher incidence of stage N1 nodal disease, which may have predisposed this cohort to disease recurrence, resulting in a lower rate of disease-free survival and subsequently reduced overall survival. However, this higher rate of nodal disease has not been seen in similar studies [11,19].

Whilst sarcopenia has been associated with a decreased rate of disease-free survival [13,19], the exact pathological mechanism underlying this observation has yet to be elucidated. Possible explanations include that skeletal muscle has been shown to regulate interleukin-6, tumor necrosis factor-alpha, and insulin resistance, and reduced muscle mass may result in a pro-inflammatory state that can dampen the host immune response to cancer [23]. Additionally, skeletal muscle produces a variety of myokines that may suppress cancer progression by promoting apoptosis, decreasing cancer cell proliferation, limiting migration, and preventing metastasis [24].

Sarcopenic patients were more likely to be restricted in their diet due to esophageal obstruction, which required pureed or enteral feeds. This finding may suggest that despite early nutritional intervention, the oral intake of these patients is inadequate to meet the increased metabolic demands that accompany esophageal cancer. However, instead of being causative, this finding may reflect that sarcopenia is associated with more advanced esophageal cancer. Supporting the latter hypothesis are previous studies that demonstrated enteral feeding to have benefits to weight maintenance, surgical complications, and lower overall mortality rates [25,26]. Interestingly, Pery et al. found that obstructing esophageal cancers at diagnosis were associated with poorer overall survival outcomes [27].

Previous larger studies have shown an association between sarcopenia and worse postoperative complications, particularly respiratory complications and anastomotic leaks [12,19]. The increased incidence of pulmonary complications observed in other studies is expected to be caused by the sarcopenic weakening of the muscles responsible for respiration and swallowing, heightening the risk of developing complications such as atelectasis, pleural effusions, aspiration, and pneumonia [12]. Although within this study no significant differences were found in the rate of postoperative complications, this finding may be attributable to the relatively small sample size.

The length of hospital stay was longer for sarcopenic patients than for the non-sarcopenic group (24 days vs. 15 days). Fehrenbach et al. also found that sarcopenic patients required longer stay in the hospital after esophagectomy (32 days vs. 19 days) [28]. Previous studies have attributed this to overall frailty and an increased incidence of postoperative complications, which extends patients' hospital stays [29].

To combat the effects of sarcopenia, some studies have investigated a program of prehabilitation to preoperatively optimize patients’ physical, psychological, and nutritional status. Halliday et al. found that a tailored program of nutrition and exercise significantly preserved skeletal muscle in cancer patients undergoing esophagectomy [8]. A recent meta-analysis of prehabilitation in esophagectomy showed that it was associated with a decrease in postoperative pulmonary complications [30]. However, this effect was only seen in observational studies and was not replicated in the randomized controlled trials; however, there was significant heterogeneity in the studies included in the analysis, which may have had a confounding effect.

The limitations of this study are its single-center recruitment and retrospective design, which may limit its wider applicability. There was some heterogeneity in the treatments patients underwent in this study, including the type of surgery, the type of neoadjuvant therapy, and a minority who did not receive neoadjuvant treatment. Although individually these differences did not reach statistical significance, their influence as a potential source of bias cannot be excluded. Further evaluation of patients' postoperative treatment, including if they received adjuvant treatment, may also further elucidate the factors that influence patients' long-term outcomes. As an open surgical technique was utilized in this study, it may be difficult to generalize these findings to patients who undergo laparoscopic/thoracoscopic surgery. Another limitation was that the number of patients included in this study was low, particularly for female patients, which necessitates further evaluation with a larger sample size.

## Conclusions

In this study, patients with preoperatively diagnosed sarcopenia on CT scans were found to have a lower overall and disease-free survival and an increased length of hospital stay when compared with non-sarcopenic patients. Sarcopenic patients also had a higher likelihood of requiring pureed or enteral feeds preoperatively. As preoperative CT staging scans for patients with esophageal cancer are ubiquitous and routine, the information required to determine patients’ sarcopenic status is already available and may assist clinicians in anticipating which patients are at higher risk of poor postoperative outcomes and which patients may benefit from a program of prehabilitation. Currently, the main barrier to the implementation of widespread preoperative sarcopenic assessment is the time-intensive process of determining if a patient is sarcopenic. Future research should focus on streamlining the process of sarcopenic assessment, in addition to further understanding the effects of sarcopenia on short- and long-term postoperative outcomes.
